# Icaritin ameliorates RANKL-mediated osteoclastogenesis and ovariectomy-induced osteoporosis

**DOI:** 10.18632/aging.205068

**Published:** 2023-10-03

**Authors:** Jun-ming Huang, Zhe Wang, Guo-Bin Qi, Qi Lai, A-lan Jiang, Yue-Qi Zhang, Kun Chen, Xiu-Hui Wang

**Affiliations:** 1 Department of Orthopedics, Shanghai University of Medicine and Health Sciences Affiliated to Zhoupu Hospital, Shanghai, China; 2 The Orthopedic Hospital, The First Affiliated Hospital of Nanchang University, Nanchang University, Nanchang, Jiangxi, China; 3 Department of Orthopedic Surgery, Zhongshan Hospital, Fudan University, Shanghai, China; 4 Medical Innovation Center, The First Affiliated Hospital of Nanchang University, Nanchang, Jiangxi, China; 5 Department of Orthopedics, The First Affiliated Hospital of USTC, Division of Life Sciences and Medicine, University of Science and Technology of China, Hefei, Anhui, China

**Keywords:** icaritin, osteoclast, RANKL, osteoporosis, mitochondria

## Abstract

A rapidly aging society and longer life expectancy are causing osteoporosis to become a global epidemic. Over the last five decades, a number of drugs aimed at reducing bone resorption or restoring bone mass have been developed, but their efficacy and safety are limited. Icaritin (ICT) is a natural compound extracted from anti-osteoporosis herb *Epimedium* spp. and has been shown to inhibit osteoclast differentiation. However, the molecular mechanism by which ICT weaken RANKL-induced osteoclast differentiation has not been completely investigated. Here, we evaluated the anti-osteoclastogenic effect of ICT *in vitro* and the potential drug candidate for treating osteoporosis *in vivo*. *In vitro* study, ICT was found to inhibit osteoclast formation and bone resorption function via downregulating transcription factors activated T cell cytoplasm 1 (NFATc1) and c-fos, which further downregulate osteoclastogenesis-specific gene. In addition, the enhanced mitochondrial mass and function required for osteoclast differentiation was mitigated by ICT. The histomorphological results from an *in vivo* study showed that ICT attenuated the bone loss associated with ovariectomy (OVX). Based on these results, we propose ICT as a promising new drug strategy for osteoporosis that inhibits osteoclast differentiation.

## INTRODUCTION

Osteoporosis has been recognized as the silent disease of the 21st century because of progressive microarchitectural deterioration of the bone tissue without any obvious symptoms or signs before the fragility fracture occurs. According to pathogenesis, osteoporosis is generally classified into two major categories, that is primary osteoporosis and secondary osteoporosis. Regarding primary osteoporosis, postmenopausal osteoporosis and senile osteoporosis are the two most common forms, the former of which occurs when the amount of estrogen secretion decrease and the latter of which is caused by specific biological aging of skeleton system [1]. Secondary osteoporosis has been demonstrated its association with various congenital diseases and endocrine disharmony, as well as nutritional status and some medications. Clinically, both men and women may develop osteoporosis. However, women are more susceptible to osteoporosis than men due to estrogen depletion during menopause which leads to decline in bone formation and increase in bone resorption activity. Furthermore, for individuals aged 50 years and over, one in two women will suffer from an osteoporotic fracture compared to one in four men [2, 3]. In light of an aging population worldwide, osteoporosis, particularly postmenopausal osteoporosis, will inevitably increase in medical and socioeconomic burden, so the prevention and treatment of this disease are of significant importance.

As we all know, bones undergo a constant remolding process mainly involving bone resorbing osteoclasts and bone forming osteoblasts to maintain skeletal size, shape, and structural integrity and regulate mineral homeostasis [4]. In normal condition, the differentiated state and function of osteoblasts and osteoclasts are in a delicate balance thus bone mass remains relatively constant. But in some medical conditions, such as menopause [5], hyperthyroidism [6], long-term glucocorticoids usage [7], and heavy metals exposure [8], the differentiation and function of osteoclasts will greatly overwhelm those of osteoblasts, which lead to reduced bone mass and strength, and eventually result in osteoporosis and fragility fracture. As early as 1950s, estrogen was proposed as pharmacological treatment for osteoporosis due to its inhibitory effect on osteoclasts, and twenty years later, anti-resorptive agent bisphosphonates were first prescribed for the treatment and prevention of osteoporosis [9]. In the following 50 years, a lot of novel pharmacological agents have been tested, only a very small part of them were finally approved to prevent and treat osteoporosis, most of which were anti-resorptive agents, including selective estrogen receptor modulators (SERMs), calcitonin, and monoclonal antibodies specific to RANKL (denosumab). Despite these drugs used for treating osteoporosis are efficient, limitations still remain. For instance, bisphosphonates are commonly used medications for osteoporosis treatment to reduce risk of fracture, but long-term usage may bring about the concern of atypical femur fractures and osteonecrosis of the jaw with limited therapeutic effect [10]. Moreover, other anti-resorptive agents were reported to induce venous thromboembolism (VTE), breast cancer, stroke, and hypercholesterolemia in osteoporosis patients [11]. To treat osteoporosis effectively without severe side-effects, new pharmacological agents are therefore urgently needed.

The traditional Chinese medicine (TCM) is one of the oldest health care systems originated from China, and has long been practiced by oriental countries for thousands of years [12]. It has been demonstrated that many herbs alleviate bone loss in bone metabolic diseases as well as promote bone healing in fractures. The herb *Epimedii*, the dried aerial parts of *Epimedium* spp., is one of widely used herbs in osteoporosis treatment formulas. Modern medical studies have demonstrated that *Epimedii* contains several kinds of flavonoid compounds and the most abundant ingredient is icariin that possesses anti-osteoporosis effect via inhibiting osteoclastogenesis and regulating osteogenic differentiation [13–15]. However, some scholars proposed that bioavailability of icariin in human body is very limited. After being ingested in body, icariin can be deglycosylated into glycoside icariin I and rhamnoside icariin II by intestinal microflora, and then aglycone, icaritin (ICT), and demethylicariin can be obtained by further removing sugar moieties [16]. In a randomized controlled trial, Yong et al. demonstrated that ICT was the main metabolites detected in serum when *Epimedium* prenylflavonoid extracts were consumed for 6 weeks [17]. In our previous study, ICT and icariin have similar effects on inhibiting osteoclast differentiation [18]. However, the molecular mechanism by which ICT weakens RANKL-induced osteoclast differentiation has not been completely investigated. In this study, we aim to explore the effect and potential mechanism of ICT on osteoclast differentiation and function by using murine bone marrow macrophages (BMMs), and to examine the therapeutic outcome of ICT on postmenopausal osteoporosis by ovariectomized mouse model.

## MATERIALS AND METHODS

### Reagents

Recombinant Murine cytokines (RANKL and M-CSF) were provided by Bio-Techne R&D systems (Minneapolis, MN, USA), which were dissolved in 0.1% BSA yielding stocking solution (100 ng/μl) and kept at −80°C before being used. Icaritin (ICT; C_21_H_20_O_6_; MW: 368.385) was manufactured by Target Molecule Corp. (Boston, MA, USA), prepared as stocking solution by dissolving in DMSO (Dimethyl sulfoxide). The Cell Counting Kit-8 (CCK-8) was also manufactured by Target Molecule Corp. (Boston, MA, USA). The cell culture reagents including minimum essential medium eagle-alpha modification (α-MEM) medium, penicillin/streptomycin, and fetal bovine serum (FBS) were bought from Gibco (Grand Island, NY, USA). The Actin-Tracker and DAPI were purchased from Beyotime (Shanghai, China). The Tartrate Resistant Acid Phosphatase (TRAP) kits, DMSO, and other common reagents of the highest purity available were gained from Sigma-Aldrich (St. Louis, MO, USA).

### BMMs isolation and culture

As reported previously, the primary bone marrow cells were isolated from the long bone marrow cavities of 4–6 weeks old mouse by flush and then culture in non-treated plates with α-MEM for 3 days. Subsequently, the BMMs were washed and harvested for further experiments [19]. In all experiments, M-CSF (30 ng/ml) was used to maintain the proliferation of BMMs.

### Cell viability assay

The harvested BMMs were seeded into 96-well plate at the same density of about 5 × 10^3^ cells/well with 30 ng/mL and cultured for 24 hours. Initially, the BMMs were cultured with higher dose of ICT (1, 5, 10, 20, and 40 μM) for 3 days to determine the IC_50_ of ICT on BMMs. Then, the BMMs were treated with vehicle (DMSO) and lower dose of ICT (0.01, 0.1, and 1 μM) for 1 day, 3 days, 5 days, and 7 days. The absorbance of 450 nm was measured by FlexStation 3 (Molecular Device, Shanghai, China) according to instruction manual of CCK-8 kit. In addition, the BMMs cultured for 7 days were subjected to LIVE/DEAD assay via protocol of LIVE/DEAD Cell Imaging Kit (R37601) (Thermo Fisher Scientific, Waltham, MA, USA). Briefly, the component A was mixed component B to create working solution, which was further added to plate for 15 mins incubation at darkroom. The live and dead cells were showed green fluorescence and red fluorescence respectively.

### Osteoclast differentiation and TRAP staining

The harvested BMMs were seeded into 96-well plate at the same density about 1 × 10^4^ cells/well with 30 ng/mL and cultured for 24 hours. Then, the BMMs were cultured in osteoclastogenic medium containing 10% FBS, 1% penicillin/streptomycin, M-CSF (30 ng/mL), and RANKL (50 ng/mL) with vehicle (DMSO) or indicated dose of ICT (0.01, 0.1, and 1 μM). In time-course assay, the 1 μM ICT were supplemented in medium at either day 1 or day 3. The fresh medium was changed every two days until osteoclasts formed, the TRAP staining was carried out by TRAP kit. The TRAP-positive cells with three or more nuclei were recognized as mature osteoclasts, which were captured by Olympus IX-71 (Olympus, Tokyo, Japan) and quantified by ImageJ software.

### F-actin ring formation evaluation

The harvested BMMs were seeded into Corning Osteo Assay Surface plate (Corning Incorporated Life Science, Corning, NY, USA) at the same density about 2 × 10^4^ cells/well with 30 ng/mL and cultured for 24 hours. After that, the BMMs were cultured in osteoclastogenic medium mentioned above. When the formed osteoclasts were observed, cells were fixed with 4% paraformaldehyde (PFA) for 20 mins and permeabilized with 0.1% (v/v) Triton X-100 for 5 mins. After that, we used Actin-Tracker and DAPI to label F-actin and nucleus respectively. The images of F-actin ring were acquired by Olympus IX-71 (Olympus, Tokyo, Japan).

### Bone resorption assay

The BMMs were cultured in 6-well plates in density of 2.5 × 10^3^ cells/cm^2^ and treated with M-CSF (30 ng/mL) and RANKL (50 ng/mL) to perform osteoclast differentiation. The formed osteoclasts were digested and seeded on Corning Osteo Assay Surface plate (Corning Incorporated Life Science, NY, USA) at the same density about 2 × 10^3^ cells/well with osteoclasts induction medium in the presence of vehicle (DMSO) or indicated dose of ICT (0.01, 0.1, and 1 μM). After 3 days culture, the osteoclasts were removed by 5% sodium hypochlorite treatment and the bone resorption pits images were captured by Olympus IX-71 (Olympus, Tokyo, Japan) and quantified by Image J.

### RNA isolation and quantitative RT-PCR

The RNA isolation and Quantitative RT-PCR were performed as described previously [18]. Briefly, the isolated BMMs were seeded on 6-well plate at density of 2.5 × 10^3^ cells/cm^2^ and cultured with M-CSF (30 ng/mL). Twenty-four hours later, the osteoclastogenic medium containing vehicle (DMSO) or ICT (1 μM) was used to induce osteoclast differentiation. Three days later, the total RNA was extracted using RNA-Quick Purification Kit (YISHAN Bio-Tec, Shanghai, China) according to the instructions. Next, cDNA was synthesized using 1 μg of extracted RNA according to instrument of Prime Script RT reagent kit (TaKaRa Biotechnology, Shiga, Japan). The synthesized cDNA was used as templates to perform qRT-PCR using SYBR (TaKaRa Biotechnology, Shiga, Japan). The GAPDH was selected as the housekeeping gene to normalize the relative expression of target genes. The specific primer sequences are as follows: GAPDH: F 5′-AGGTCGGTGTGAACGGATTTG-3′ and R 3′-TGTAGACCATGTAGTTGAGGTCA-5′; NFATc1: F 5′-GACCCGGAGTTCGACTTCG-3′ and R 3′-TGACACTAGGGGACACATAACTG-5′; c-fos: F 5′-CGGGTTTCAACGCCGACTA-3′ and R 3′-TTGGCACTAGAGACGGACAGA-5′; RANK: F 5′-GGACGGTGTTGCAGCAGAT-3′ and R 3′-GCAGTCTGAGTTCCAGTGGTA-5′; Cathepsin K: 5′-GAAGAAGACTCACCAGAAGCAG-3′ and R 3′-TCCAGGTTATGGGCAGAGATT-5′; MMP9: F 5′-CTGGACAGCCAGACACTAAAG-3′ and R 3′-CTCGCGGCAAGTCTTCAGAG-5′; TRAP: F 5′-CACTCCCACCCTGAGATTTGT-3′ and R 3′-CATCGTCTGCACGGTTCTG-5′.

### Western blot analyses

The harvested BMMs were seeded into 6-well plates at the same density about 2.5 × 10^3^ cells/cm^2^ with M-CSF (30 ng/mL). Followed by overnight culture, the BMMs were cultured in osteoclastogenic medium containing vehicle (DMSO) or ICT (1 μM) for 3 days. According to the process mentioned previously [20], the whole protein was prepared by treating cells with RIPA lysis buffer containing broad spectrum phosphatase inhibitors and PMSF, whose concentration was determined by the BCA protein assay kit (Thermo Fisher Scientific, Waltham, MA, USA), and 10 μg proteins was following electrophoresed on 10% SDS-polyacrylamide gel and transferred to the PVDF membranes (Millipore, Burlington, MA, USA). After blocking with 5% non-fat milk at room temperature for 1 hour, the membranes were incubated with specific primary antibodies at 4°C overnight. Then, membranes were washed with TBS-Tween and were incubated with corresponding HRP-conjugated secondary antibodies for 1 hour at room temperature. Lastly, the protein signals were visualized using electrochemical luminescence reagent (ECL) (Millipore, Burlington, MA, USA) on Bio-Rad system and analyzed using Image J software.

The following antibodies were provided by Abmart (Shanghai, China): NFATc1 (TD6446), c-fos (T56596), Cathepsin K (TD6614), MMP9 (TA5228), Parkin (T56641), DRP1 (TD7037), MFN2 (T56638), FIS1 (TD12005). The other antibodies were provided by Proteintech Group (Rosemont, IL, USA): GAPDH (60004-1-Ig). The secondary antibodies, including HRP conjugated anti-rabbit or anti mouse antibody were provided by Jackson ImmunoResearch Inc. (West Grove, PA, USA).

### Mitochondrial membrane potential detection

The harvested BMMs were seeded into 6-well plates at the same density about 2.5 × 10^3^ cells/cm^2^ with M-CSF (30 ng/mL) and cultured for 24 hours. Then, the BMMs were cultured in osteoclastogenic medium with vehicle (DMSO) or ICT (1 μM). Three days later, the JC-1 staining solution (No. 10009172) (Cayman Chemical, Ann Arbor, MI, USA) were added at rate of 100 μl per ml. After incubated in CO_2_ incubator at 37°C for 20 mins, the mitochondrial membrane potential was detected by fluorescence microscope.

### Transmission electron microscopy (TEM)

The harvested BMMs were seeded into 6-well plates at the same density about 2.5 × 10^3^ cells/cm^2^ with M-CSF (30 ng/mL) and cultured for 24 hours. Then, the BMMs were cultured in osteoclastogenic medium with vehicle (DMSO) or ICT (1 μM). Three days later, cells were fixed with 2.5% glutaraldehyde, postfixed with 1% osmium tetroxide, dehydrated in a graded series of ethanol, and embedded in resin. Next, the embedded samples were cut into 60 nm sections by an ultramicrotome (Leica EM UC7). After being stained with uranyl acetate and lead citrate, the sections were finally observed at TEM (HT7800/HT7700, HITACHI).

### Ovariectomy (OVX) murine model establishment

The animal experiment was designed and performed following the guide of the NIH “Principles of Laboratory Animal Care” (1996 Revised Version) and under the supervision of the Animal Use and Care Committee of Shanghai University of Medicine and Health Sciences Affiliated to Zhoupu Hospital. The wild-type C57/BL6 female mice (12-week-old) were purchased from Gempharmatech Co., Ltd (Nanjing, China) and kept in the specific-pathogen-free animal care facility. All mice were fed with sterile chow and water and kept in constant condition with controlled temperature (25 ± 2°C), humidity (60 ± 5%), and illumination (12:12 light/dark cycle.). Mice (21–25 g) were randomly arranged into three groups (*n* = 6), that is sham-operated mice with vehicle treatment (Sham), bilateral ovariectomized mice with vehicle treatment (OVX), and bilateral ovariectomized mice with ICT treatment (ICT). The treatment dose of ICT is 10 mg/kg that was referred to previous study that ICT showed no toxicity for mice [21]. The sham and OVX surgery were performed according to previous description [18, 20, 22] and the vehicle and ICT were given by intraperitoneal injections every two day for 6 weeks. All mice were sacrificed by over dose of pentobarbital, then femurs were collected for the following experiments.

### Micro-computed tomography (μ-CT) and histomorphometric analysis

After removing muscle and soft tissue, the fixed distal femur was scanned and analyzed by microcomputed tomography (μ-CT) system by following settings: 100 kV source voltage and 98 mA source current with a 10.5 mm resolution. The protocol of μ-CT and histomorphometric analysis referred to our previous study [22]. Briefly, the range at distance of 0.5 mm below subchondral bone was selected as the region of interest (ROI) for further analysis. Three-dimensional reconstruction of femur and evaluation of bone volume/tissue volume (BV/TV), trabecular number (Tb.N), trabecular thickness (Tb.Th), and trabecular separation (Tb.Sp) were all performed in system software installed in μ-CT. After μ-CT scanning, all femurs were decalcified and embedded in paraffin. The 5 μm bone sections were cut along the coronal plane on a microtome and the TRAP staining were performed. Lastly, the representative pictures of distal femur trabecular area were captured and analyzed by microscopy.

### Statistical analysis

Each experiment in this study was independently repeated more than three times and the data were described as mean ± SD. We performed one-way ANOVA with Tukey–Kramer honest significant difference (HSD) test to examine the significant differences where more than two groups existed by GraphPad Prism 7.0 software (GraphPad Software, San Diego, CA, USA) and unpaired *t*-test was used for two groups comparisons. A value of *p* < 0.05 was considered to indicate statistically significant differences.

## RESULTS

### ICT inhibits osteoclast differentiation induced by RANKL

**Figure 1:**
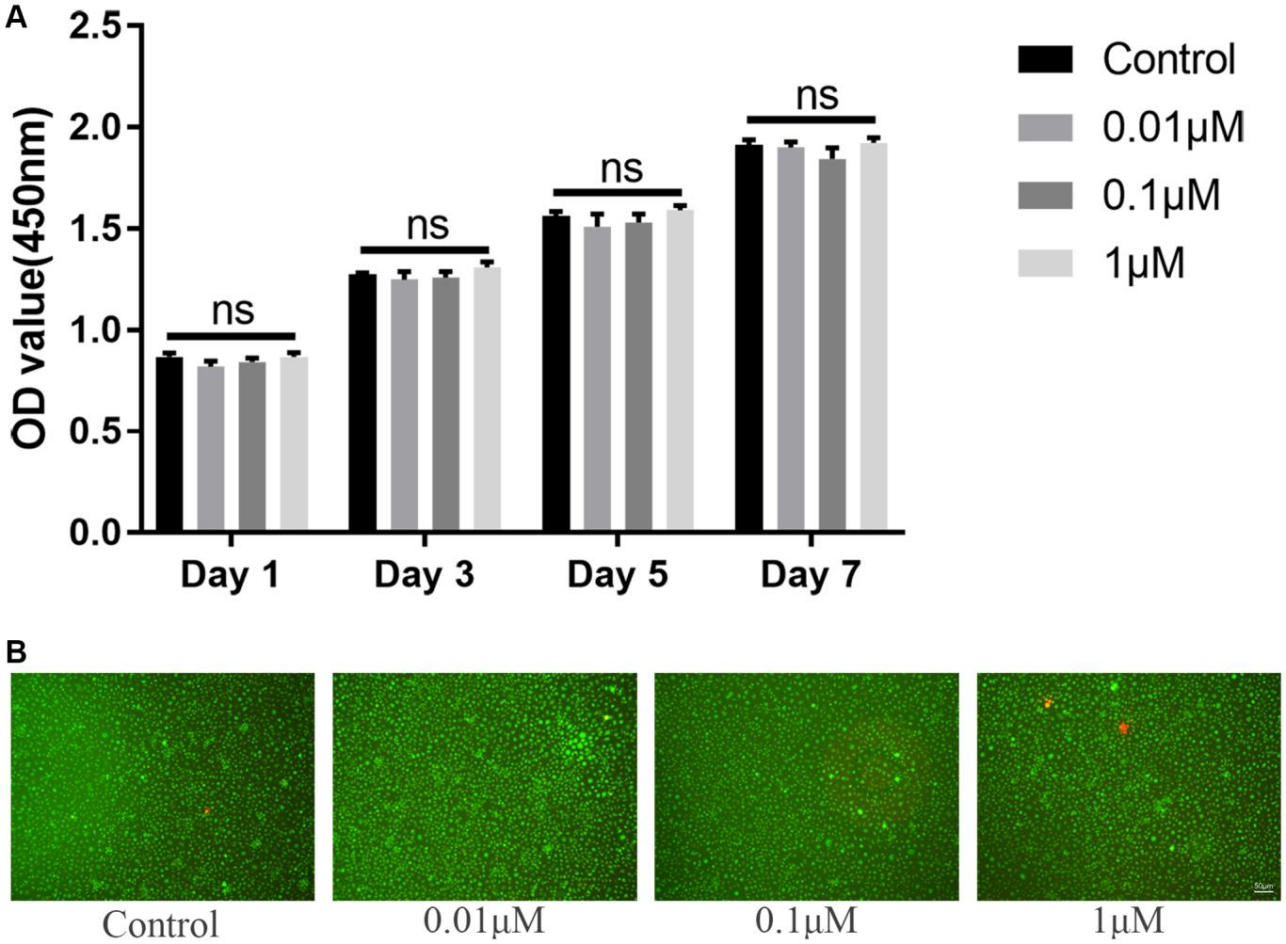
**ICT has no toxicity on BMMs.** (**A**) Cytotoxicity of ICT on BMMs at day 1, day 3, day 5, and day 7 by CCK-8 assay. (**B**) The live/dead staining of BMMs after culture with ICT for 7 days. All data were presented as mean ± SD, *n* = 3. Abbreviation: ns: not statistical significance.

**Figure 2:**
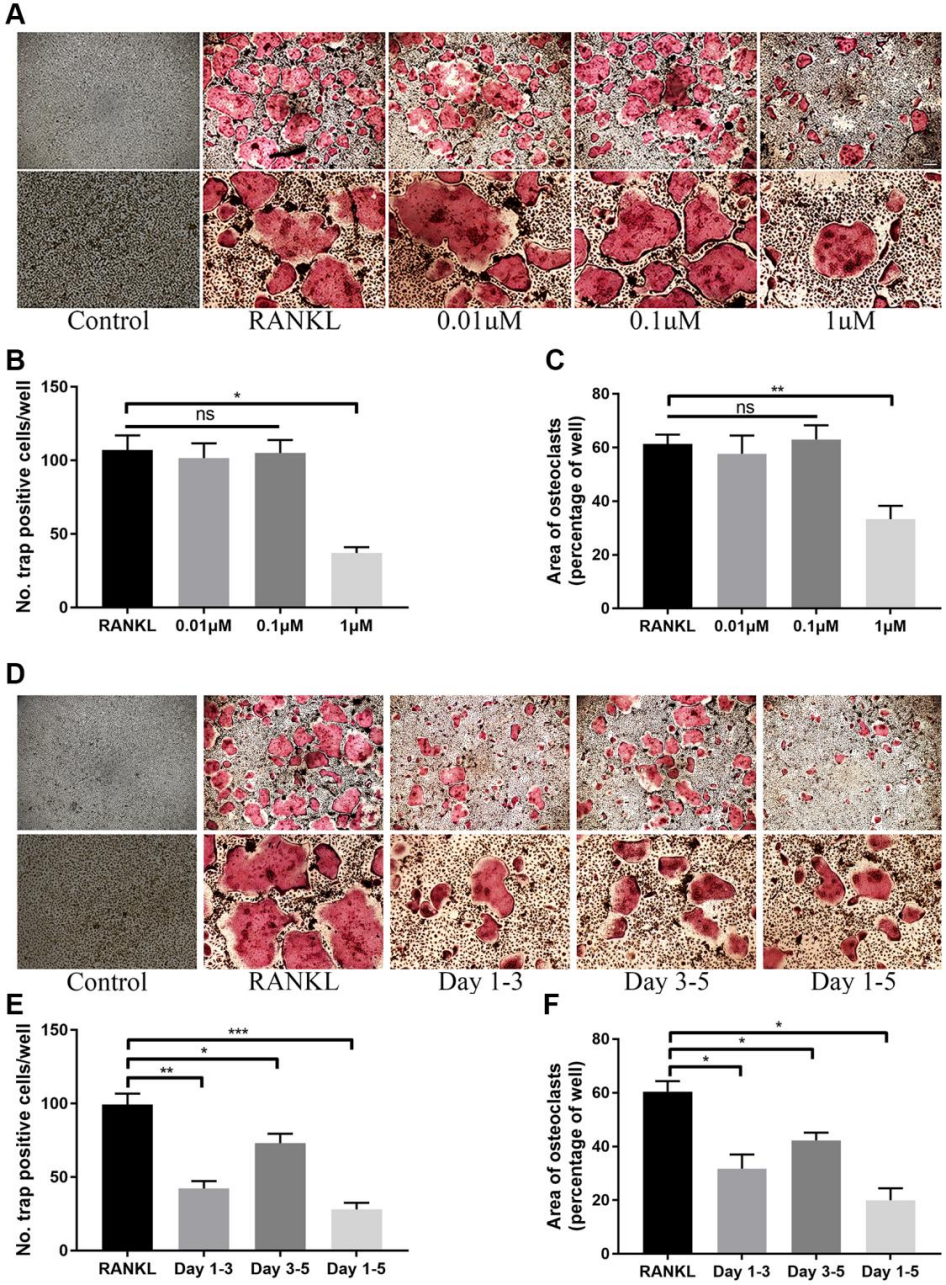
**ICT inhibits osteoclast differentiation induced by RANKL.** (**A**) Representative TRAP staining images of BMMs treated with DMSO or different doses of ICT in the presence of M-CSF and RANKL for 5 days. (**B**, **C**) Quantification of the number and area of TRAP-positive cells. (**D**) Representative TRAP staining images of BMMs treated with ICT in the presence of M-CSF and RANKL for the indicated time. (**E**, **F**) Quantification of the number and area of TRAP-positive cells. All data were presented as mean ± SD, *n* = 3. Abbreviation: ns: not statistical significance; ^*^P < 0.05; ^**^P < 0.01; ^***^P < 0.001.

*In vitro* study, we firstly evaluated the potential toxicity of ICT on BMMs via CCK-8 and LIVE/DEAD assay. Initially, for determining the IC_50_ of ICT on BMMs, BMMs were cultured with higher dose of ICT (1, 5, 10, 20, and 40 μM) for 3 days, and the results of the cell activity assay demonstrated that the IC_50_ of ICT on BMMs is 16.33 μM (Supplementary Figure 1). Then BMMs were cultured with lower dose of ICT (0.01, 0.1, and 1 μM), which will be used in following study, and CCK-8 assay showed that indicated dose of ICT has no toxicity on BMMs during continuous culture (Figure 1A). Furthermore, in live/dead assay, no obvious dead cells were observed after 7-days culture (Figure 1B). Subsequently, we investigated the effect of ICT on osteoclast differentiation. As shown in TRAP staining, ICT suppressed RANKL-induced osteoclast differentiation at concentration of 1 μM ICT and about 60% osteoclasts numbers were reduced compared to RANKL group (Figure 2A–2C). Furthermore, ICT treatment (1 μM) in the early stage (day 1–3) or in the late stage (day 3–5) of osteoclast differentiation both resulted in decreased osteoclast formation, and according to the count and size of osteoclast, the effect of ICT on early stage of osteoclast differentiation is superior to late stage of osteoclast differentiation (Figure 2D–2F).

### ICT suppresses osteoclast function

**Figure 3:**
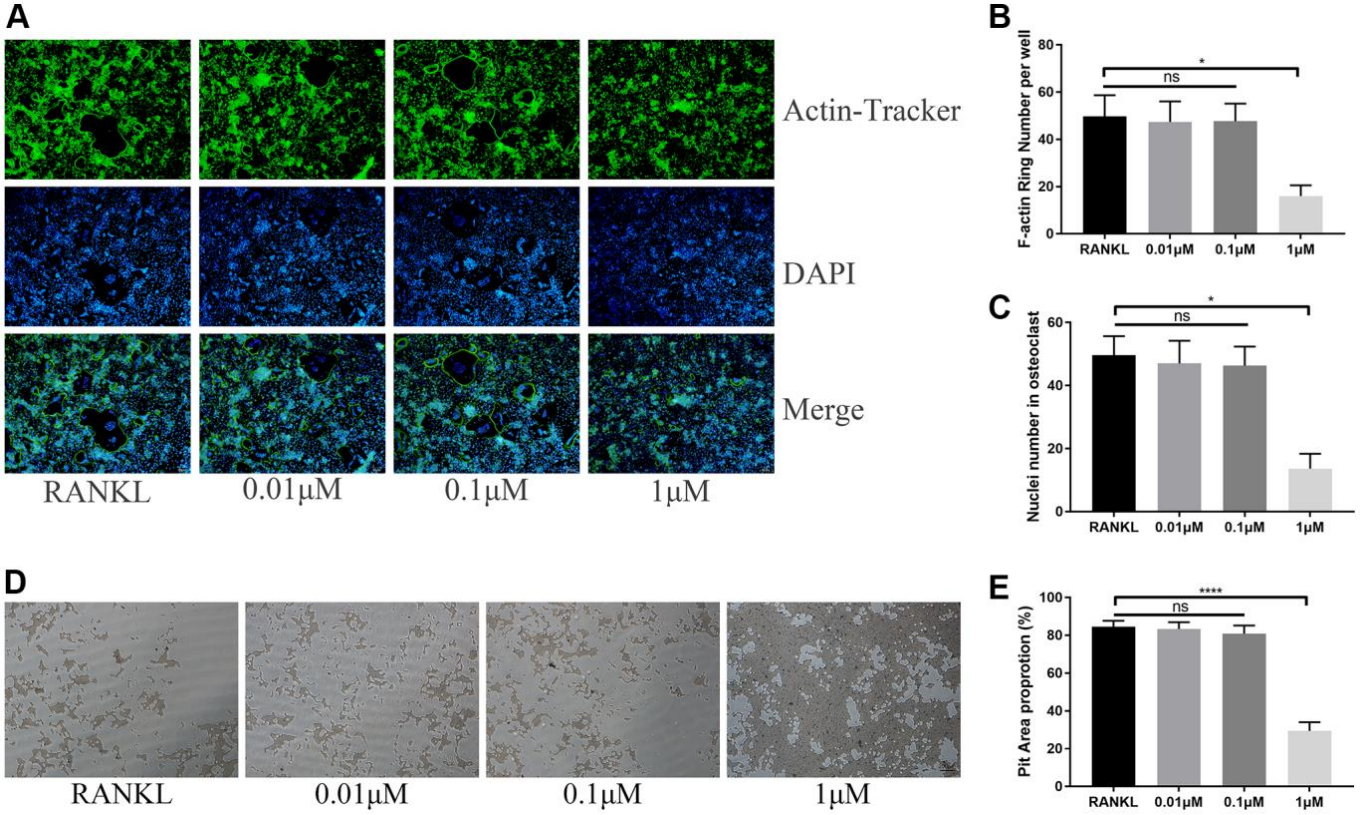
**ICT suppresses osteoclast function.** (**A**) Representative images of F-actin rings. (**B**) Quantification of the F-actin rings number. (**C**) Mean number of nuclei in each cell. (**D**) Representative images of pit area by ICT-treated osteoclast on Osseo Assay plate. (**E**) Quantification of pit area. All data were presented as mean ± SD, *n* = 3. Abbreviation: ns: not statistical significance; ^*^P < 0.05; ^****^P < 0.0001.

Due to the inhibition on osteoclast differentiation, we speculated whether ICT also inhibits osteoclastic activity. When we observed differentiated osteoclast in Corning Osteo Assay Surface plate, we used Actin-Tracker to label F-actin ring formation that is an observed trademark for osteoclast mature and provides a prerequisite for osteoclastic function and found that the formation of F-actin ring was disrupted through observing the reduced nucleation per osteoclast and F-actin count (Figure 3A–3C). Unsurprisingly, in bone resorption assay, the resorption pit corroded by osteoclasts was also decreased when formation osteoclast cultured with 1 μM ICT (Figure 3D, 3E).

### ICT suppresses osteoclast-specific protein and gene expression

**Figure 4:**
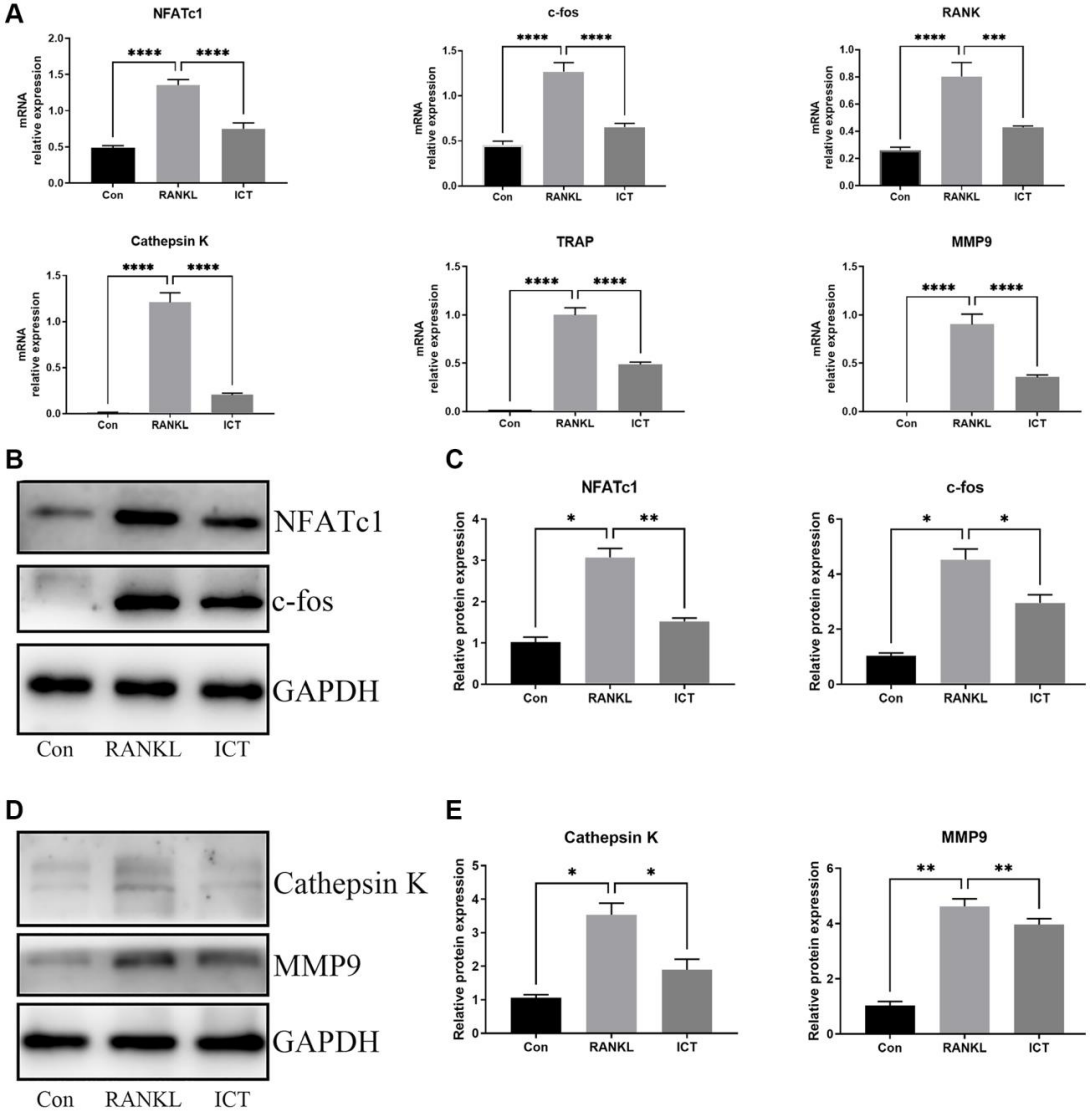
**ICT abrogates RANKL-induced NFATc1 and c-fos transcription and following osteoclast-related genes.** BMMs were cultured in osteoclastogenic medium containing vehicle (DMSO) or ICT (1 μM) for 3 days. (**A**) The relative mRNA expression of NFATc1, c-fos, RANK, Cathepsin K, MMP9, TRAP. (**B**) Representative protein bands of NFATc1 and c-fos. (**C**) Quantitative analysis of NFATc1 and c-fos. (**D**) Representative protein bands of Cathepsin K and MMP9. (**E**) Quantitative analysis of Cathepsin K and MMP9. All data were presented as mean ± SD, *n* = 3, ^*^P < 0.05; ^**^P < 0.01; ^***^P < 0.001.

For further investigating the expression of osteoclast-specific genes during osteoclast differentiation, the BMMs were cultured in osteoclastogenic medium with DMSO or ICT (1 μM) for 3 days. The specific genes responsible for osteoclast formation and osteoclastic bone resorption activity were selected, including NFATc1, c-fos, RANK, Cathepsin K, MMP9, and TRAP, and the normalized data revealed that ICT markedly attenuated RANKL-stimulated expression of NFATc1, c-fos, RANK, Cathepsin K, MMP9, and TRAP (Figure 4A). In western blot assay, the data demonstrated that the protein expression of NFATc1, c-fos, MMP9, and Cathepsin K were also reduced compared with RANKL stimulation, which is in line with the mRNA results (Figure 4B–4E).

### ICT downregulates mitochondrial mass and function

**Figure 5:**
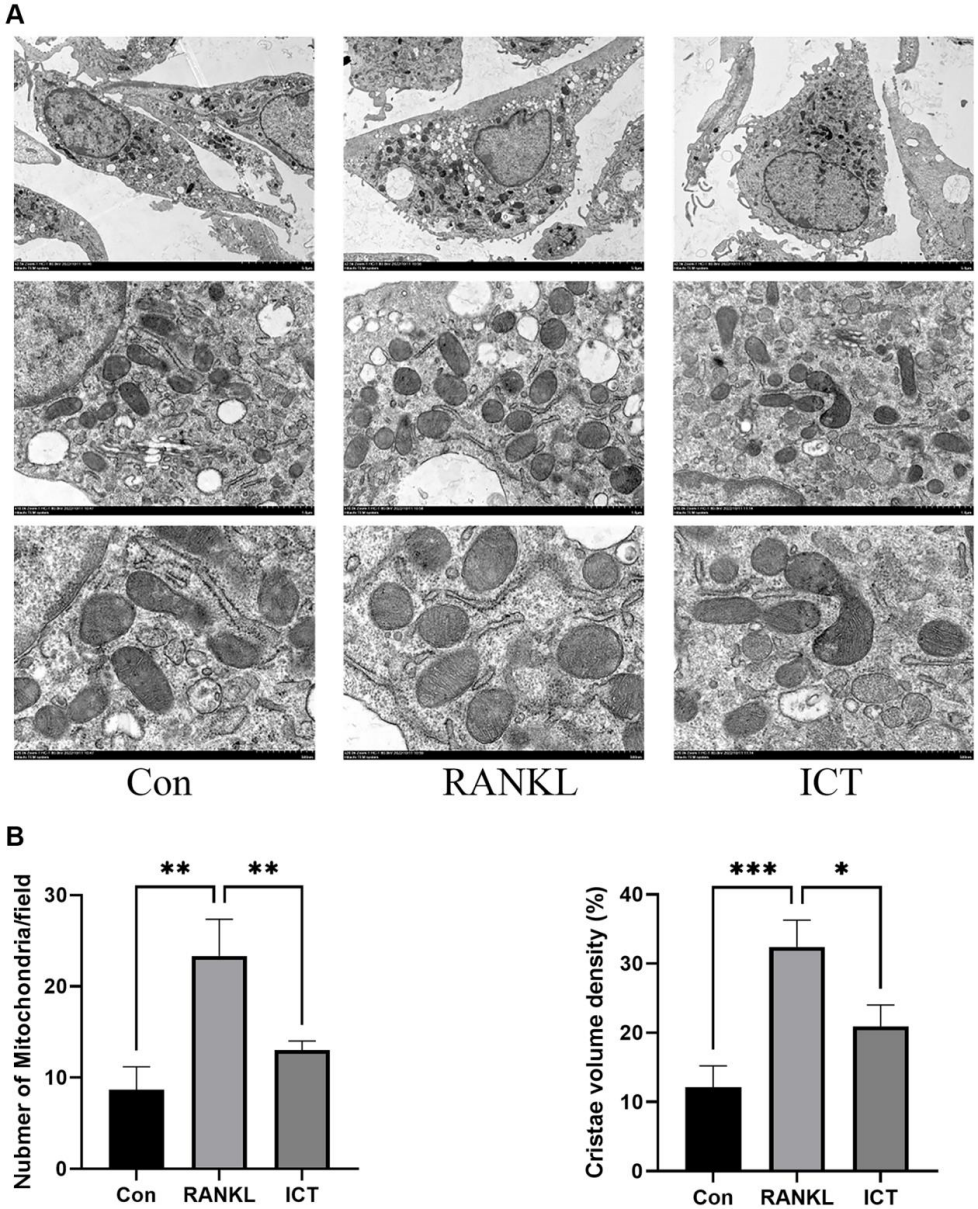
**Representative images of ultrastructural analysis of mitochondria during osteoclast differentiation.** (**A**) Representative image of mitochondria. (**B**) The quantitative analysis of mitochondria number and cristae density. All data were presented as mean ± SD, *n* = 3, ^*^P < 0.05; ^**^P < 0.01; ^***^P < 0.001.

**Figure 6:**
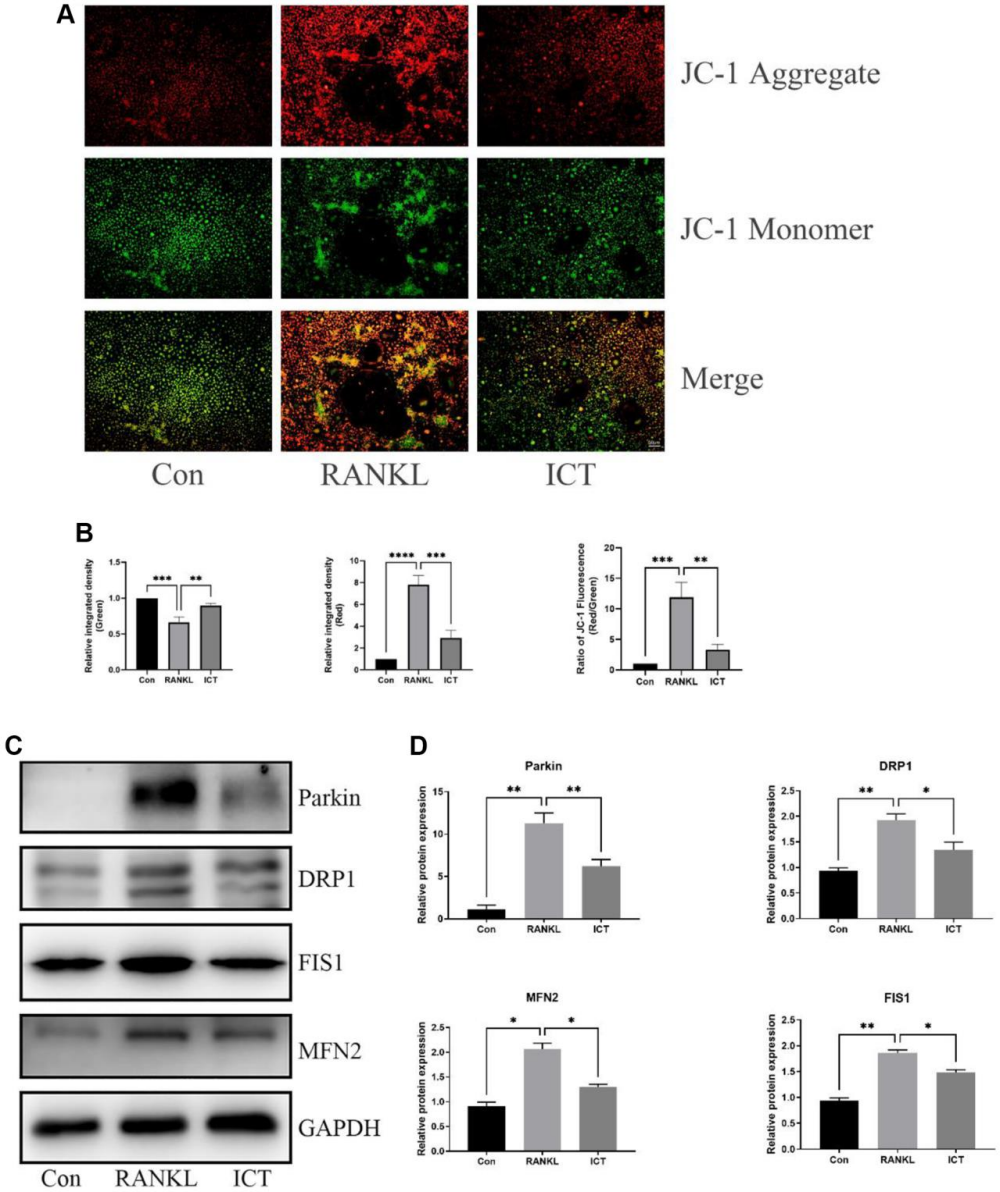
**ICT inhibited the mitochondrial membrane potential and reduced mitochondrial fusion and fission.** (**A**) Representative fluorescence photographs of mitochondrial membrane potential stained by JC-1. (**B**) Quantitative analysis of membrane potential by measuring the ratio of red and green fluorescence intensity. (**C**) Representative protein bands of Parkin, DRP1, FIS1, and MFN2. (**D**) Quantitative analysis of Parkin, DRP1, FIS1, and MFN2. All data were presented as mean ± SD, *n* = 3, ^*^P < 0.05; ^**^P < 0.01; ^***^P < 0.001; ^****^P < 0.0001.

As described previously, osteoclast differentiation and activation are energy-intensive processes, the amount and activity of mitochondria in cell will be enhanced [23]. The BMMs were cultured in osteoclastogenic medium for 3 days. Through TEM, we found that RANKL stimulation increased mitochondria mass and ICT could reduce the number of mitochondria (Figure 5A). Then, we observed the ultrastructure of mitochondria and found that the cristae in BMMs treated with M-CSF and RANKL was richer than cultured with M-CSF only, and ICT could reduce the density of cristae (Figure 5B). In addition, we detected the mitochondrial membrane potential by JC-1 staining, which is a prerequisite for the production of ATP and reflect the activity of the entire mitochondrial function [24]. When mitochondria membrane potentials are high, the JC-1 will aggregate and emit red fluorescence, otherwise, JC-1 will exist in the form of a monomer and produce green fluorescence. Our results showed that during osteoclast differentiation, the mitochondria membrane potentials was increased, which was significantly reduced after treatment with ICT (Figure 6A, 6B). Mitochondrial fusion and fission are known to regulate the function and morphology of mitochondria [25]. Therefore, we detected the protein level involved in mitochondrial fusion and fission by western-blot. As shown in our results, the level of Parkin, DRP1, MFN2, and FIS1 were upregulated when BMMs were treated with RANKL, and ICT inhibited the elevated level of Parkin, DRP1, MFN2, and FIS1 (Figure 6C, 6D). In other word, ICT inhibited the stimulated mitochondrial fusion and fission.

### ICT mitigates OVX-induced bone loss

**Figure 7:**
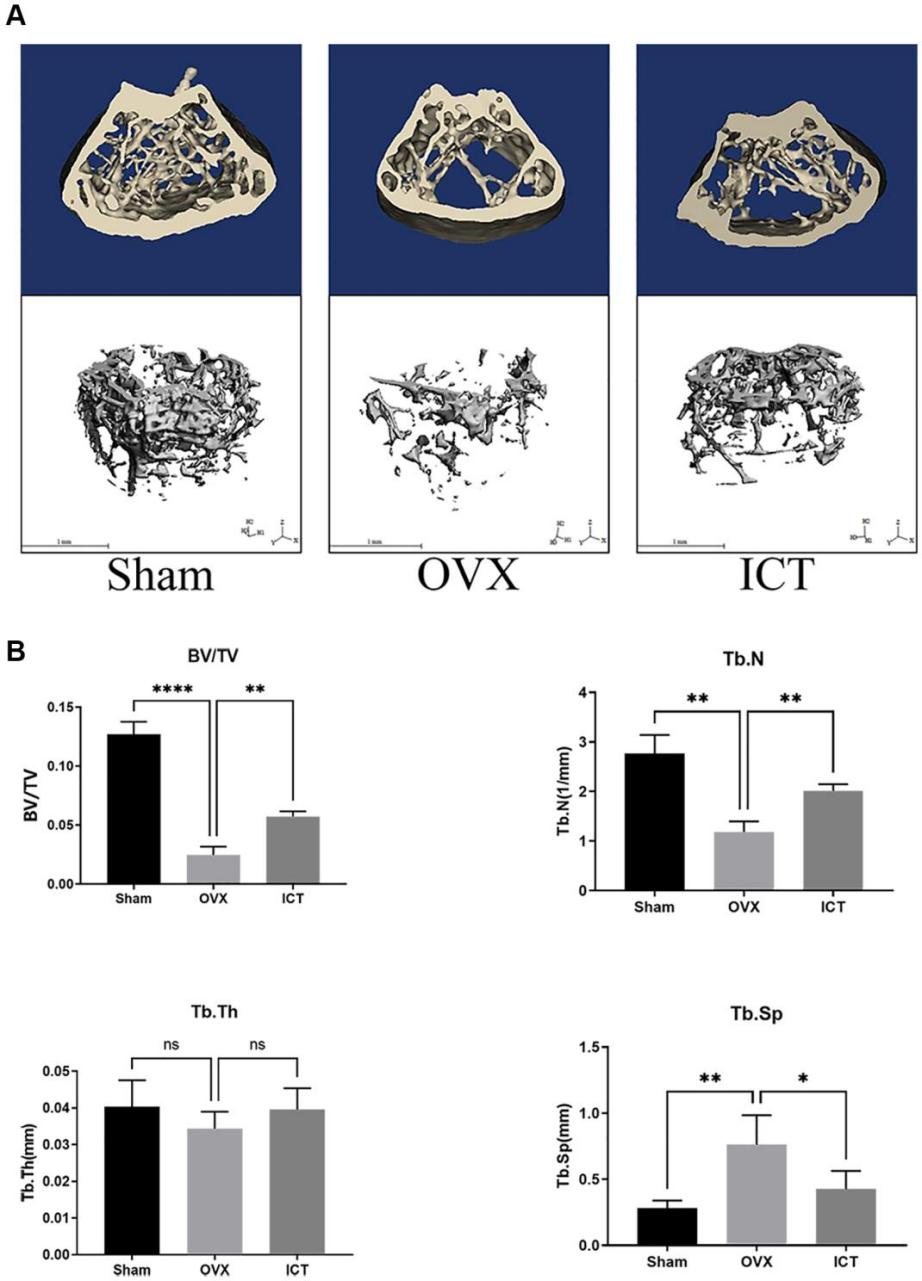
**ICT suppresses OVX-induced bone loss.** Twelve-week-old female mice were randomly arranged into three groups, that is Sham (Sham + Vehicle), OVX (OVX + Vehicle), and ICT (OVX + ICT). After being subjected to sham or OVX surgery, mice were treated with vehicle or ICT for 6 weeks. (**A**) Representative 3D reconstruction micro-CT images of the trabecular bone from each group. (**B**) The measurement data of BV/TV, Tb.Th, Tb.N, and Tb.Sp from each group through built-in software of CT system. All data were presented as mean ± SD, *n* = 6. Abbreviation: ns: not statistical significance. ^*^P < 0.05; ^**^P < 0.01; ^****^P < 0.0001.

**Figure 8:**
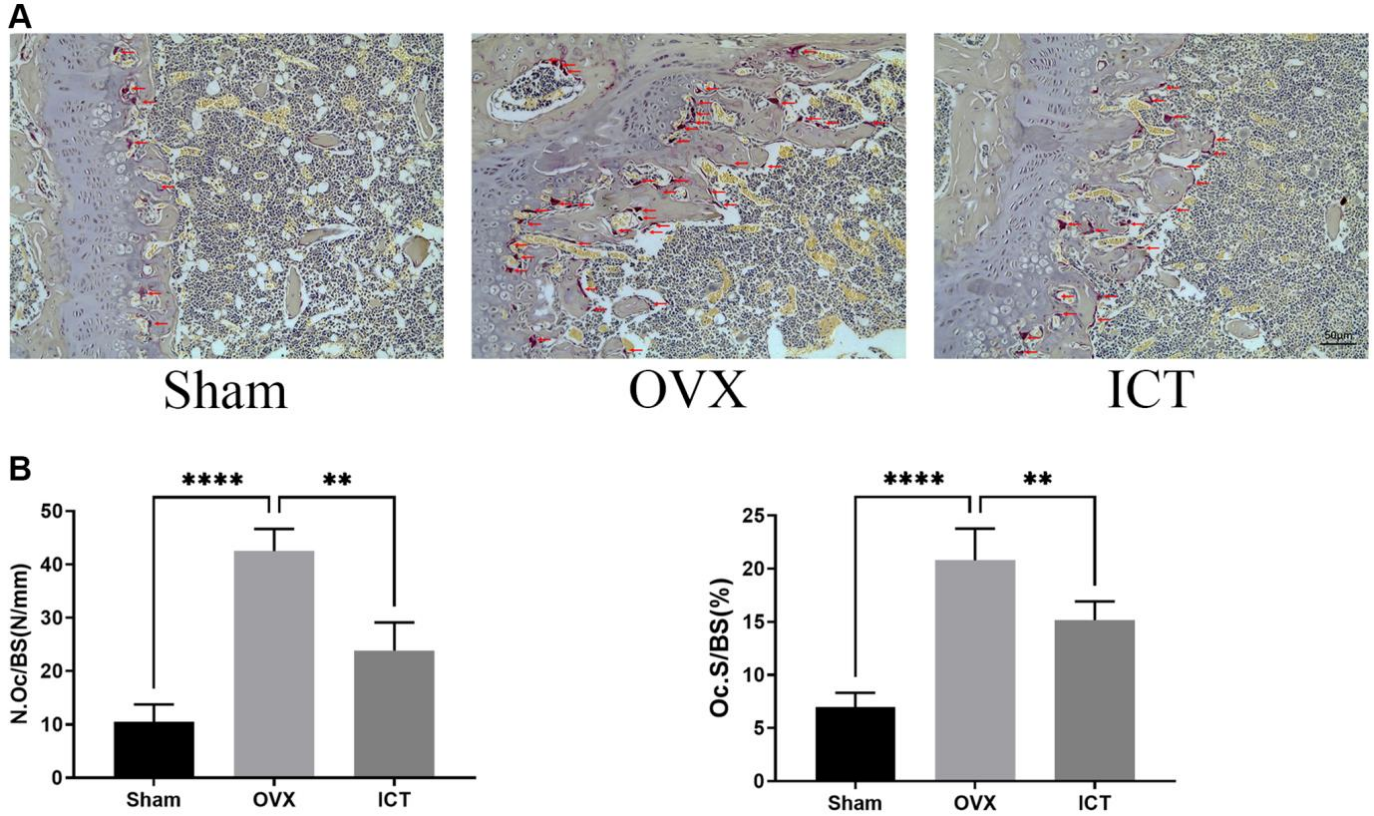
**ICT inhibits osteoclast formation *in vivo*.** (**A**) Representative TRAP staining images of femurs from each group. (**B**) The quantitative histomorphometric analysis of TRAP-positive osteoclasts, N.Oc/BS and OcS/BS. All data were presented as mean ± SD, *n* = 6, ^**^P < 0.01; ^****^P < 0.0001.

In our *vitro* study, we demonstrated that ICT administration could regulate osteoclast differentiation and osteoclastic bone resorption activity, thus we wanted to further investigate the potential therapeutic effect of ICT on osteoporosis through *in vivo* study. Through scanning the femur samples, the dramatic compromised trabecular bone mass was observed in OVX group, which was featured with decreased BV/TV, Tb.N, and elevated Tb.Sp, and ICT treatment reversed the bone loss induced by estrogen deficiency (Figure 7A, 7B). In the bone section, the results of TRAP staining indicated that the number of osteoclasts normalized to the bone surface (N.Oc/BS), and surface area of osteoclast to bone surface area (OcS/BS) were increased in distal femur and the osteoclast formation *in vivo* was inhibited when OVX mice were administrated with ICT (Figure 8A, 8B). According to the data from *in vivo* study, we concluded ICT ameliorates OVX-induced bone loss via inhibiting osteoclasts formation.

## DISCUSSION

Because osteoporotic bone loss occurs or osteoporotic bone tissue deteriorates without any obvious symptoms, osteoporosis is usually ignored by patients until the low-traumatic fracture occurs. Among many kinds of osteoporosis, postmenopausal osteoporosis is the most common type, in which the rapidity of bone resorption becomes faster than that of bone formation, thus the accelerating bone loss emerge [26]. As reported previously, the incidence of fracture in the community represents bimodal distribution with peaks in youth and in the elderly [27]. In youth, the prevalence of fracture mainly occurred in young men due to substantial trauma, but above the age of 35 years, the overall incidence of fracture in women rises sharply until the morbidity rate of female becomes twice that of male. With the aging of the global population, osteoporosis mortality is on the rise due to brittle fractures [28], osteoporosis, especially postmenopausal osteoporosis imposes a substantial medical and economic burden on society.

The osteoclasts, which are generated from monocyte-macrophage lineage precursor cells, are the primary cells responsible for bone resorption. The excessive osteoclast differentiation and activity resulting in abnormal bone resorption is the main cause for osteoporosis, which also contribute to developing control strategies and drug research for osteoporosis. In our study, we demonstrated that ICT could inhibit formation and function of osteoclasts *in vitro* which is consistent with previous study through investigating osteoclast differentiation of RAW 264.7 and human PBMC [29]. During osteoclast differentiation, the transcription factor, c-fos and NFATc1 are the master regulator, the former of which binds to NFATc1 promoter to promote NFATc1 expression in the early stage of osteoclast differentiation and acts as a stimulus to trigger osteoclast or macrophage differentiation from monocyte progenitors [29]. The mice with c-fos mutant were found to have a block in the differentiation of bone-resorbing osteoclasts that results in osteopetrotic phenotype [30]. The other transcription factor, NFATc1 is the most important regulator in osteoclast formation through regulating the expression of osteoclast-related genes, which are involved in the regulation of osteoclast differentiation, fusion and activation [31]. Once differentiation completed, mature osteoclast dissolves the bone matrix by secreting protons, and lysosomal proteases like the TRAP, MMP9, and Cathepsin K. Our study proved that ICT dramatically suppressed both mRNA and protein expression of NFATc1, cfos, MMP9, and Cathepsin K. In addition, we investigated the mitochondrial mass and function during osteoclast differentiation and confirmed that ICT reduced the mitochondria mass as well as cristae density in mitochondria, and the increased mitochondrial membrane potential and mitochondrial fusion and fission were attenuated by ICT.

As we know, cell survival and homeostasis are maintained by the hydrolysis of adenosine triphosphate (ATP), the key element for energy transfer and storage, which is synthesized by mitochondria, the “powerhouse” in eukaryotic cells [32]. Osteoclasts differentiation is an energy-consuming process supported by high metabolic activity and steadily accumulating evidences supported that the mitochondria play a key role in the intracellular signaling for maintaining osteoclast differentiation and maturation [33, 34]. Lemma and his colleague observed increased mitochondrial mass along osteoclast differentiation and the cristae structure in mature osteoclast is richer through ultrastructural analysis [23]. In our study, we observed similar change about mitochondria during osteoclast differentiation and ICT treatment could reduce mitochondrial mass and cristae density. Furthermore, appropriate mitochondrial membrane potential levels are prerequisite for the production of ATP and reflect the activity of the entire mitochondrial function [24]. We determined the effect of ICT on mitochondrial membrane potential during osteoclast differentiation and found that the enhanced mitochondrial membrane potential stimulated by RANKL was weakened after supplementation with ICT. To ensure mitochondrial morphology, membrane potential, and function are maintained, fusion and fission must be balanced in order to remove damaged mitochondria [25]. In mitochondria, DRP1 and FIS1 mediate mitochondrial fission and the damaged mitochondria are subsequently degraded by Parkin dependent mitophagy and Parkin independent mitophagy. Mitochondrial fusion occurs when two adjacent mitochondria fuse to form an elongated mitochondrion, and MFN2 facilitates this process. As a result of RANKL induction, mitochondrial fusion and division of BMMs were stimulated, while ICT treatment significantly reduced these events. Thus, we concluded that osteoclast differentiation was accompanied by increased mitochondrial mass, membrane potential, and fusion and fission, which were reduced as ICT was added.

In addition, we should note that there is a certain limitation that cannot be ignored in our study. The inhibitory effect and underlying mechanism of ICT on osteoclast differentiation had been primarily explored, but the specific target of ICT on osteoclast differentiation remains unclear, it is necessary to perform further in-depth study to identify the binding substances of ICT for better understanding its role in osteoclastogenesis.

As a conclusion of our study, we demonstrated that ICT inhibited osteoclast differentiation by impairing mitochondrial function and mass. Furthermore, ICT administration could attenuate bone loss induced by estrogen deficiency. Collectedly, our investigations provide new insight regarding potential use of ICT for osteoporosis and osteoclast-related bone metabolic disorders therapy.

## Supplementary Materials

Supplementary Figure 1
