# Supplementary material for: Icaritin ameliorates RANKL-mediated osteoclastogenesis and ovariectomy-induced osteoporosis
Source: Aging (Albany NY). 2023 Oct 3;15(19):10213–36. doi: 10.18632/aging.205068 (PMC10599742; doi:10.18632/aging.205068)
Supplement: Supplementary Figure 1 [file aging-15-205068-s001.pdf]

SUPPLEMENTARY FIGURE

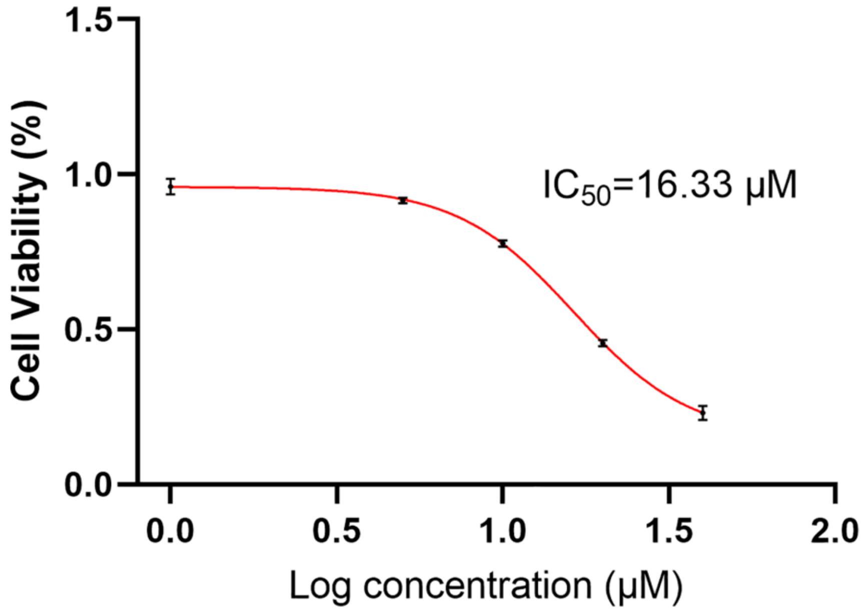

Supplementary Figure 1. The IC_{50} concentrations of icaritin detected in BMMs.
